# Phomopsin-A and Quinolizidine Alkaloids Concentrations in *Lupinus albus* Seeds: Effect of Aqueous and Gaseous Ozone Application

**DOI:** 10.3390/foods15020326

**Published:** 2026-01-15

**Authors:** Francesco Buccioni, Chiara Rossi, Annalisa Serio, Sara Palmieri, Fabiola Eugelio, Antonello Paparella

**Affiliations:** Department of Bioscience and Technology for Food, Agriculture and Environment, University of Teramo, Via R. Balzarini 1, 64100 Teramo, Italy; fbuccioni@unite.it (F.B.); aserio@unite.it (A.S.); spalmieri@unite.it (S.P.); feugelio@unite.it (F.E.); apaparella@unite.it (A.P.)

**Keywords:** lupin, phomopsin-A, *Diaporthe toxica*, mycotoxin, sustainability

## Abstract

Recent studies on novel protein sources unveiled lupins as a promising substitute for meat consumption. However, lupin cultivation and processing include significant safety concerns, such as quinolizidine alkaloids (QAs) and the possible growth of toxigenic fungi as *Diaporthe toxica*, which produces the mycotoxin phomopsin-A (PHO-A). Therefore, this study aims to assess the influence of gaseous and aqueous ozone on lupin beans as environmentally sustainable methods for detoxifying QAs and PHO-A mycotoxins, thereby addressing both these safety challenges. Three distinct aqueous and gaseous ozone treatments (4, 6, and 8 h, at 7.00 ppm O_3_ concentration) were applied on lupin seeds inoculated with *D. toxica* DSM 1894. A good effectiveness of aqueous O_3_ in the reduction in PHO-A (about 20%) was demonstrated, independently of the treatment duration, along with the reduction in some QAs typically encountered in lupin. Additionally, a significant reduction in *D. toxica* count was observed after 4 h treatment with aqueous O_3_. In contrast, results for gaseous O_3_ treatments did not show any significant effectiveness on either PHO-A or QAs. Conversely, none of the treatments applied significantly affected lupin color. In conclusion, aqueous ozone treatment demonstrated significant potential for the reduction in PHO-A and QAs, and the insights acquired from this work may aid in mitigating the dangers associated with lupin intake. Nevertheless, additional research is required to cover current knowledge gaps. Specifically, toxicological assays on PHO-A degradation by-products or O_3_ combination with other hurdles is required to enhance treatments and preserve lupins’ nutrients.

## 1. Introduction

In recent times, the world has been facing multiple crises that unequivocally affect food production. Climate change, pandemics, and geopolitical conflicts, as well as the rapid increase in population, have led to an imbalance between supply and demand, and a disruption of national and international food supply chains has already started [1,2]. Specifically, the demand for new nutritious food sources is rising, especially those that are vegetable-based, due to the extremely negative impact that the livestock industry has on global warming and environmental pollution [3]. Among these foods, legumes, edible insects, macroalgae, and cultured meat are being assessed as the most reasonable alternatives to meat, because of their smaller environmental impact and valuable nutritional and health benefits [4,5]. Legumes have historically been consumed by humans, with evidence from various regions indicating their inclusion in early human diets approximately 12,000 years ago [6]. Nonetheless, their consumption has surged in recent years due to their sustainability compared to animal protein sources.

Among legumes, lupins (*Lupinus* spp.) emerge as major players in vegetable-based food research, due to their great nutritional value and high protein content, even higher than the largely used soybeans. In fact, despite varying between species and cultivars, crude protein in lupin account for 31 to 52% of dry matter, with exceptionally high concentrations of lysine, leucine, and phenylalanine [7]. The *Lupinus* genus includes more than 400 species, of which *L. albus* (white lupin), *L. angustifolius* (blue lupin), *L. luteus* (yellow lupin), and *L. mutabilis* (Andean lupin) are the most cultivated. In spite of the high yield, lupin cultivation areas and application remain relatively small, due to the lack of studies on crop practices [8].

Despite their potential, lupin also represents a concern for human health due to the presence of antinutritional factors and its external contamination risks. One of the most important issues is linked to the presence of quinolizidine alkaloids (Qas). Qas are cyclic, nitrogen-containing secondary metabolites of the lupin plant that can cause poisoning in humans by affecting cardiovascular and nervous systems [9]. Secondly, numerous studies have shown that lupins are highly susceptible to the presence of phomopsin A (PHO-A), a mycotoxin characterized by a cyclic hexapeptide structure produced by the fungus *Diaporthe toxica* (anamorph *Phomopsis leptostromiformis*) that has lupin as the main host. PHO-A is responsible for severe toxicity in numerous animal species. This suggests a possible risk also for human exposure [10]. Therefore, to ensure the safe use of lupin beans and expand their food applications, it is crucial to develop approaches that effectively mitigate both these classes of undesirable compounds.

Currently, the reduction in Qas concentration in lupins intended for human consumption involves a post-harvest processing procedure known as ‘debittering’. Lupin seeds are first immersed in water for 24 h to facilitate moisture absorption. Thereafter, the rehydrated seeds are cooked at 95 °C for 25 min and then washed several times to promote alkaloid extraction through the exchange of substances between seeds and water. Throughout this procedure, a food-grade acid (e.g., lactic acid) is typically added to reach a pH below 4.5 that hinders microb”al g’owth. While this debittering process is currently the most common and effective method for removing alkaloids in the food industry, it requires large volumes of water and therefore causes unsustainable resource consumption and substantial economic losses.

Based on these challenges, alternative and more sustainable detoxification methods are deemed necessary. Ozone (O_3_) is a strong oxidizing agent that has already attracted the attention of the scientific community for its preservation properties, as documented in prior studies, and recently garnered interest from the food industry as a potential method for food disinfection. In fact, it has demonstrated significant effectiveness against a wide range of microorganisms and mycotoxins affecting foods [11]. Particularly, O_3_ showed the ability to reduce aflatoxins up to 91%, as well as deoxynivalenol and ochratoxins even with less efficacy [12]. In addition, O_3_ rapidly auto-decomposes into molecular oxygen, leaving no residues neither in the treated food nor the environment, representing a “green” alternative to classical chemicals used for disinfection. Although its mechanisms of action are not completely clear yet, some studies hypothesized that it could react with functional groups in the cell membrane or in the mycotoxin chemical structure [12,13]. Moreover, recent studies demonstrated that O_3_ treatments can exert a positive effect on ascorbic acid retention in vegetables and, consequently, have no impact on antioxidant potential of species such as lupin and cause only minor structural changes in protein and starch molecules [14,15]. Furthermore, O_3_ showed notable results in reducing the levels of secondary metabolites across different vegetable species, including alkaloids, even though the published data remain very limited [16].

To the best of our knowledge, no studies have yet examined the effectiveness of O_3_ treatments in lowering PHO-A or Qas. Therefore, this study aimed to develop and evaluate sustainable processing methods for enhancing the safety and quality of *Lupinus albus* beans. Specifically, the first aim was to investigate the effectiveness of aqueous and gaseous ozone treatments in reducing PHO-A content, by assessing various application methods and exposure times. Moreover, the study explored the efficacy of the same ozone treatments in reducing Qas levels.

## 2. Materials and Methods

### 2.1. Fungal Strain

The fungal toxigenic strain *Diaporthe toxica* DSM 1894, anamorph *Phomopsis leptostromiformis*, was purchased from the Leibniz Institute (DSMZ-German Collection of Microorganisms and Cell Cultures GmbH, Braunshweig, Germany). In accordance with the institute’s recommendations, the strain was routinely cultivated on Oat Flake Medium (OFM), using the following preparation method: 30 g of dehydrated oat flakes were boiled under stirring in 500 mL of distilled water for 10 min; then, the volume was filled up to 1 L, and 20 g agar were added. Successively, the medium was sterilized at 121 °C for 20 min and shaken before pouring into sterile Petri dishes.

### 2.2. Lupin Samples Inoculation

*Lupinus albus* L. var. Multitalia beans from Southern Italy were kindly provided by Madama Oliva SpA. Lupins were artificially inoculated as optimized in previous studies [17], according to the following procedure: dry lupin beans were sterilized at 121 °C for 15 min to prevent any potential contamination from naturally occurring microbial species. Then, lupin beans were immersed into sterile water at a 1:2 *w*/*v* ratio under agitation for 30 min, to obtain a 0.98 ± 0.01 a_w_, measured by an Aqualab 4TE hygrometer (METER Group, Inc., Pullman, WA, USA), which is one of the optimal conditions for fungal growth and mycotoxin production [18]. Round mycelial plugs of 8 mm diameter were inoculated into the center of Water Agar (WA) Petri dishes. Then, 10 rehydrated lupins were distributed encircling the inoculum at 35 mm distance to ensure equal and simultaneous inoculation. Finally, the plates were incubated at 25 ± 1 °C to ensure an optimal growth of the fungus. The time when the hyphae reached the beans was considered the initial time point (t0) of the experiment; from t0, WA plates containing lupin beans were kept incubated at the same temperature for 168 h.

**Figure 1:**
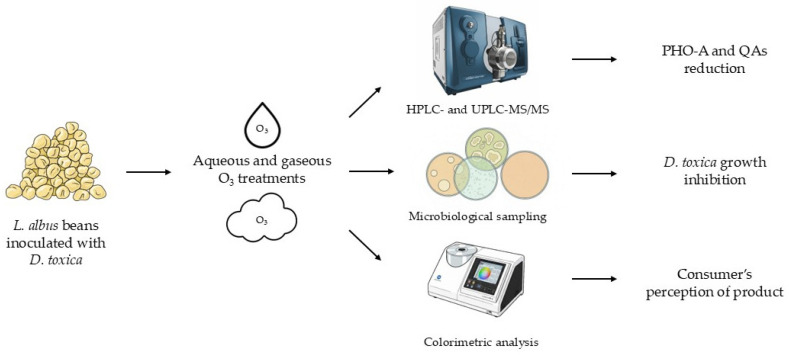
Flowchart of the experimental procedures.

Following the incubation period, contaminated lupin beans were collected and analyzed for PHO-A and Qas concentration, *D. toxica* counts, and color parameters, as detailed in Figure 1.

### 2.3. Ozone Treatment

O_3_ treatments were performed both in water and in gaseous phases, using a 15 g/h O_3_ generator (Biofresh S.r.l., San Lazzaro di Savena, Bologna, Italy) equipped with an ozone analyser model UV-100 (Interlink Electronics, Inc., Camarillo, CA, USA) for gaseous treatment, and a programmable logic controller (PLC) (B&C Electronics S.r.l., Carnate, MB, Italy) for the aqueous treatment. These devices allowed a proportional, integrative, and derivative control of O_3_ generation, dispersed O_3_ concentration, and temperature by means of an electrode and a probe, respectively. O_3_ was generated in a self-assembled, cubical Plexiglas chamber of 65 cm edge dimension. For gaseous O_3_ treatment, the chamber was left empty, while for aqueous treatment it was filled with 100 L tap water.

**Figure 2:**
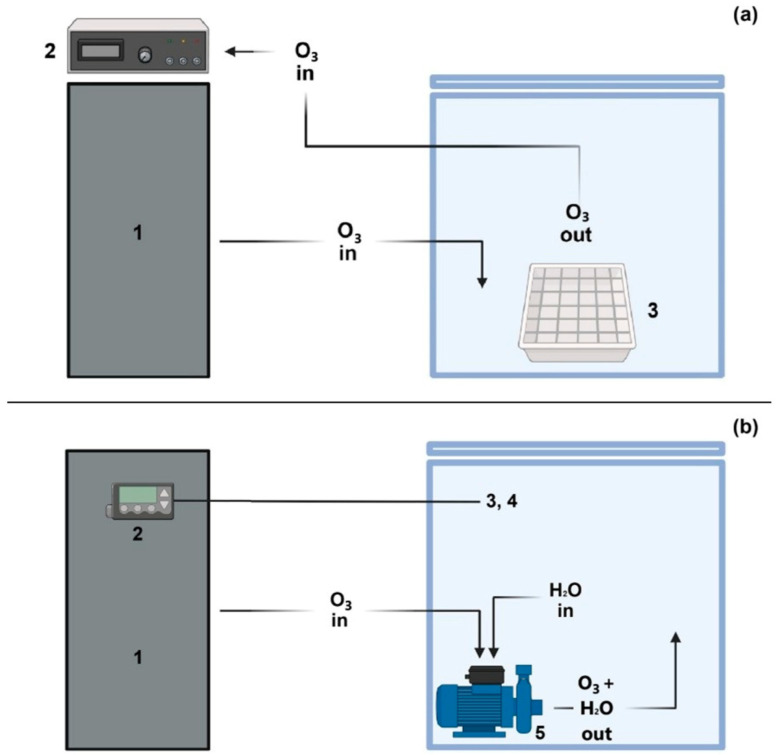
Plant configuration for (**a**) gaseous O_3_ treatment (1—generator; 2—O_3_ analyser; 3—stainless steel stand) and (**b**) aqueous O_3_ treatment (1—generator; 2—PLC; 3, 4—electrode and probe; 5—pump).

One hundred inoculated lupin beans were prepared for each analysis. For the treatments with gaseous O_3_, lupin beans were posed on a stainless-steel mesh stand, placed at the bottom of the chamber (Figure 2), whereas the treatments with aqueous O_3_ were performed by immersing lupin beans in water and maintaining them under agitation by means of a pump that was also used to dissolve O_3_ in water in the chamber (Figure 2).

After some preliminary trials at 1.5, 3.0, and 3.5 ppm O_3_ concentration, performed according to the literature [19], where neither PHO-A nor alkaloids concentration reduction was observed, the treated samples were subjected to a continuous O_3_ flow at a concentration of 7.00 ± 0.01 ppm for alternatively 4, 6, or 8 h. The time of analysis was calculated from the stabilization of O_3_ concentration in the chamber. For each treatment, a control (C) sample was considered. Control sample for gaseous ozone treatment was produced by leaving the same number of lupin beans in the chamber without O_3_ for the same time of the treatment.

Instead, C samples for aqueous ozone treatment were obtained by immersing one hundred inoculated lupin beans under agitation into the corresponding water volume and time, with no O_3_ dispersion. Temperature of the chamber was monitored by the PLC device and maintained always constant at 25.0 ± 0.1 °C. Tests were performed in triplicate. Samples and treatments are described in Table 1.

### 2.4. PHO-A Quantification in Lupin Samples

The quantification of PHO-A was performed according to Eugelio et al. [20], by the following method: 200 mg lupin samples, were milled with a domestic blender and reduced to a slurry immediately after O_3_ treatment, and extracted with an 80:20 (*v*/*v*) CH_3_CN/H_2_O solution employing a Precellys Evolution homogenizer (Bertin Technologies, Montigny-Le-Bretonneux, France). The extracts were furtherly centrifuged at 11,424 rcf, and 500 µL supernatant was collected. The extraction solution was evaporated with a SpeedVac Concentrator (Thermo Fisher Scientific, Waltham, MA, USA), and the precipitate was resuspended into 500 µL H_2_O with CF_3_COOH 0.1%. The resulting samples were filtered through Amicon Ultra 0.5 Centrifugal Filters 3K devices (Merck KGaA, Darmstadt, Germany) and further purified by µ-Solid Phase Extraction (SPE) clean-up. After extraction, PHO-A in lupin samples was determined by an ACQUITY UPLC H-Class System (Waters Corp., Milford, CT, USA) coupled to a 4500 Qtrap mass spectrometer (Sciex, Toronto, ON, Canada), equipped with an electrospray ionization (ESI) source. The chromatographic run was carried out by using an Excel 2 C18-PFP (100 × 2.1 mm) column from ACE (Aberdeen, UK), packed with 2 µm particles and equipped with a security guard column, while the mobile phases consisted of H_2_O 5 mM HCOONH_4_ (A) and ACN/MeOH 50:50 (*v*/*v*) with 0.1% HCOOH (B). The UHPLC-MS/MS method for PHO-A quantification on lupin samples was validated in accordance with the SANTE/11312/2021 guidelines for analytical quality control and method validation procedures for pesticide residues analysis in food and feed [21], following the approach described by Eugelio et al. [20]. Validation included the evaluation of linearity, carry-over, recovery, matrix effect, limits of detection (LOD), and quantification (LOQ), as well as accuracy and precision.

### 2.5. Qas Quantification in Lupin Samples

Lupanine, sparteine, multiflorine, and hydroxylupanine (OH-lupanine) in the samples were quantified by processing lupin beans according to Eugelio et al. [9]. The samples were mechanically homogenized, and an aliquot of 200 mg was extracted with 1 mL MeOH/H_2_O 60:40 (*v*/*v*) solution by Precellys Evolution homogenizer. Then, the samples were subjected to centrifugation at 11,424 rcf, and 50 µL supernatant were collected and diluted to 1 mL to obtain a final H_2_O/MeOH ratio equal to 90:10 (*v*/*v*). This solution was purified by performing a clean-up with polymeric SPE cartridges. Analysis of QA was performed with an ACQUITY UPLC H-Class System (Waters Corp., Milford, MA, USA) coupled with a 4500 Qtrap mass spectrometer (Sciex, Toronto, ON, Canada), equipped with a heated ESI source. The analytes were separated using an Excel 2 C18-PFP (10 cm × 2.1 mm ID) column from ACE (Aberdeen, UK), packed with 2 µm particles and equipped with a security guard column. The mobile phases consisted of H_2_O with 0.1% of heptafluorobutyric acid (HFBA) (C) and ACN/MeOH 50:50 (*v*/*v*) with 0.1% of HFBA (D). The UHPLC-MS/MS method for Qas quantification on lupin samples was validated according to the Food and Drug Administration (FDA) guidelines [22], following the procedure previously described by Eugelio et al. [9]. The validation included the assessment of linearity, carry-over, recovery, matrix effect, LOD, LOQ, accuracy, and precision.

### 2.6. Microbiological Analysis

To evaluate the effect of O_3_ treatment on the *D. toxica* count on lupins, microbiological sampling was carried out as follows: 25 g inoculated lupin beans were homogenized at 1:10 ratio with physiological saline solution (0.90% NaCl) for 5 min at 230 rpm using a peristaltic homogenizer (Stomacher Lab Blender 400 Circulator, Seward, UK). The obtained suspension was serially diluted 1:10 and plated onto sterile Yeast, Peptone, Dextrose (YPD) agar plates. *D. toxica* counts were evaluated after 72 h incubation at 25 °C. A sterile culture medium was used as a blank control to confirm all operations were carried out in aseptic conditions. Tests were conducted in triplicate.

### 2.7. Color Determination of Lupin Samples

To analyze the effect of ozone on lupins, their color change has been evaluated as the most important sensory attribute of the product for consumer acceptance. Lupin beans color analysis was performed on uninoculated samples subjected to the same treatments of the inoculated ones, by recording CIELab color space coordinates L* (lightness), a* (redness), b* (yellowness), C (chroma), and h (hue angle), by means of a CR-5 colorimeter configured with D65 standard illuminant and 10° standard observer angle (Konica Minolta Sensing Europe, Tokyo, Japan), equipped with a CM-A195 3 mm diameter target mask (Konica Minolta Sensing Europe, Tokyo, Japan). These coordinates were selected to render lupin color in a uniform space before and after treatments and evaluate whether a numerical change in values could identify a perceived difference for the consumer. To this effort, the global color difference was calculated as follows: ΔE = ((L*_T_ − L*_C_)^2^ + (a*_T_ − a*_C_)^2^ + (b*_T_ − b*_C_)^2^)^1/2^, and a* and b* values were used to calculate hue angle h° = arctan (b*/a*) and chroma C = (a*^2^ + b*^2^)^1/2^ [23]. Perceivable differences in color were classified according to Adekunte et al. [24]. Values were acquired through reflectance measurements of samples. Reported values represent the values of 5 lupin beans from each sample. Five measurements were performed on different areas of each lupin bean.

### 2.8. Data Statistical Analysis

Software Analyst 1.7.2 and Multiquant 3.0.3 were used to collect and process data and quantify PHO-A and alkaloids in lupin samples, respectively. Statistical data analysis was performed using GraphPad Prism 8.0.2 (GraphPad Software, Inc., Boston, MA, USA). *t*-tests, or analysis of variances followed by either Dunnett’s or Tukey’s tests (*p* < 0.05) were used to determine whether O_3_ treatment caused significant differences in PHO-A, Qas concentration, fungal counts and color parameters between each treatment and its corresponding control, and among different treatments.

## 3. Results

### 3.1. PHO-A Analysis

The first considerations regard the effect of O_3_ treatments on phomopsin A content. In detail, quantification of PHO-A revealed the efficacy of aqueous O_3_ in reducing the mycotoxin on lupin seeds, starting from roughly 50 ppm of the control samples.

**Figure 3:**
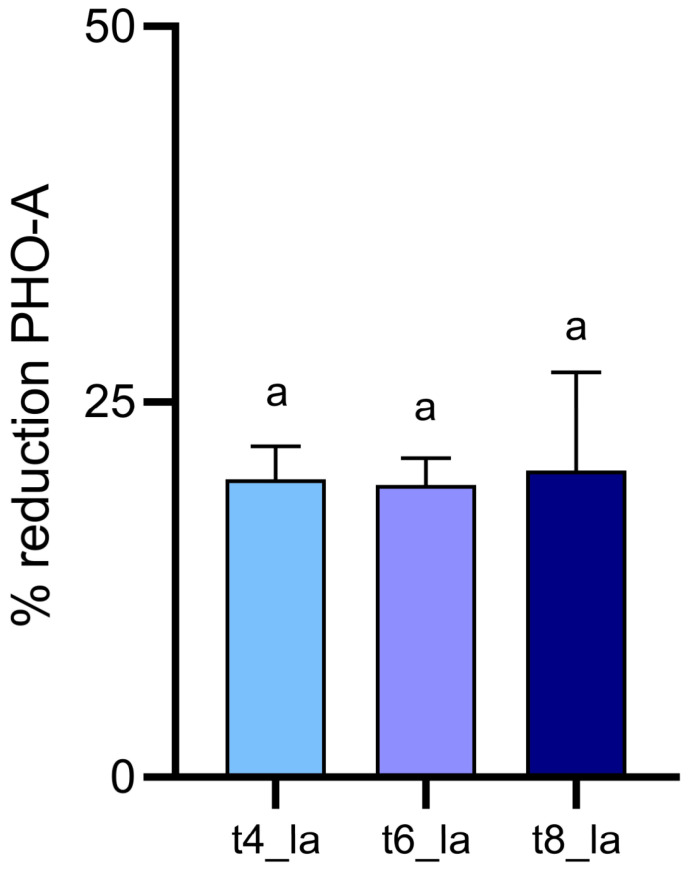
Reduction percentages of PHO-A in lupin beans samples treated with an O_3_ concentration of 7.00 ± 0.01 ppm in comparison to control. T4_la: 4 h aqueous treatment; t6_la: 6 h aqueous treatment; t8_la: 8 h aqueous treatment. Different lowercase letters above samples columns represent significant differences (*p* < 0.05) among samples.

Particularly, exposure to aqueous O_3_ allowed to achieve approximately 20% detoxification of PHO-A in the different treatments, with similar results in the different samples (Figure 3). In fact, lupin beans subjected to 4 h treatment showed a decrease in PHO-A content of 19.83%; after 6 h, 19.44% reduction was achieved, while 8 h treatment produced a 20.40% reduction. Nonetheless, no significant differences were observed among these samples (*p* > 0.05). Conversely, the administered gaseous O_3_ treatments did not diminish PHO-A levels, regardless of the duration of application.

### 3.2. Qas Analysis

**Figure 4:**
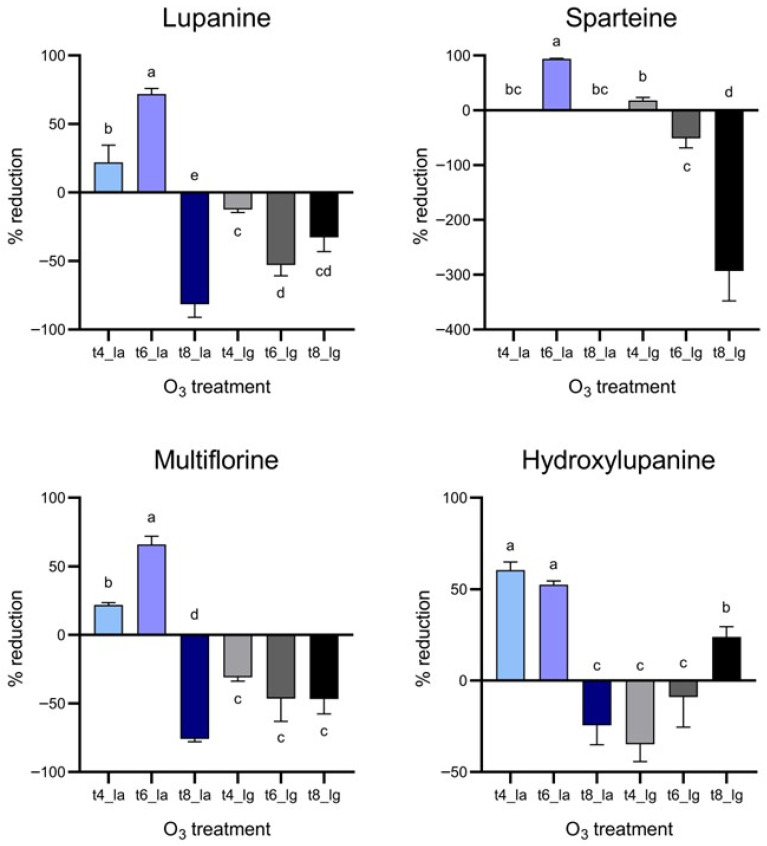
Reduction percentages of lupanine, sparteine, multiflorine and OH-lupanine in lupin beans samples treated with an O_3_ concentration of 7.00 ± 0.01 ppm, in comparison to Control sample. T4_la: 4 h aqueous treatment; t6_la: 6 h aqueous treatment; t8_la: 8 h aqueous treatment; t4_lg: 4 h gaseous treatment; t6_lg: 6 h gaseous treatment; t8_lg: 8 h gaseous treatment. Different lowercase letters above samples columns represent significant differences (*p* < 0.05) among samples.

Figure 4 shows the effectiveness of the different O_3_ treatments in reducing the alkaloid content in the spiked lupins. Specifically, it should be noted that negative reduction values represent an increase in alkaloid concentration. In particular, the main and most common four alkaloids in lupins were investigated. As it can be observed, treatments effectiveness was strongly dependent on the alkaloid chemical structure. In detail, considering lupanine, extended treatments (from 4 h to 6 h) with aqueous O_3_ improved the percentage of reduction from 22.17% to 71.86%. Nevertheless, lupanine did not decrease when lupins were treated by gaseous O_3_. Concerning spartein, the effect of the aqueous treatment was only evident after 6 h treatment, since in the other samples the concentration was below the limit of detection (LOD). However, the same treatment caused an almost complete reduction, ranging from 0.167 ppm to 0.021 ppm.

The gaseous O_3_ treatment showed an effectiveness inversely proportional to its duration, starting from the reduction in spartein after 4 h treatment, up to an increase (indicated by a negative reduction value) after 8 h. Multiflorine followed a behavior similar to lupanine, while increasing of roughly 75% after 8 h. In contrast, OH-lupanine exhibited a completely different trend: in fact, considering aqueous O_3_, the longer the treatment lasted, the less effective it was in reducing the alkaloid concentration; instead, the gaseous O_3_ treatment effectiveness was proportional to the duration of the treatment itself, allowing a reduction of 23.99% after 8 h.

### 3.3. Microbiological Analysis

Microbiological investigations were performed to assess the possibility of reducing the initial *Diaporthe toxica* count whether a direct reduction in PHO-A or alkaloids was not possible.

**Figure 5:**
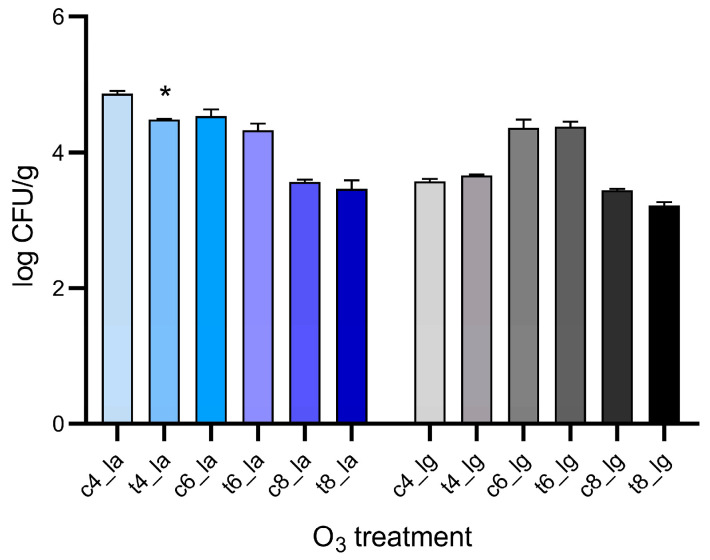
Fungal load in control (c samples) and in lupin beans samples treated with an O_3_ concentration of 7.00 ± 0.01 ppm (t samples). Samples nomenclature refers to that listed in Table 1. *p*-values for significant differences between control and treated samples are summarized with * *p* < 0.05.

The data obtained indicated that O_3_ treatments resulted in relatively minimal reduction in fungal growth. In addition, the only significant reduction was observed after a 4 h treatment in aqueous O_3_, in which *D. toxica* counts decreased from 4.86 log CFU/g to 4.48 log CFU/g. In detail, although a decrease in log CFU/g was obtained in almost every case, these variations were not significant (Figure 5). The minor fluctuations observed across the different treatments, such as the 6 h gaseous ozone treatment, have to be considered consistent with the inherent biological variability associated with natural fungal colonization of lupin samples.

### 3.4. Color Evaluation of Lupin Samples

**Figure 6:**
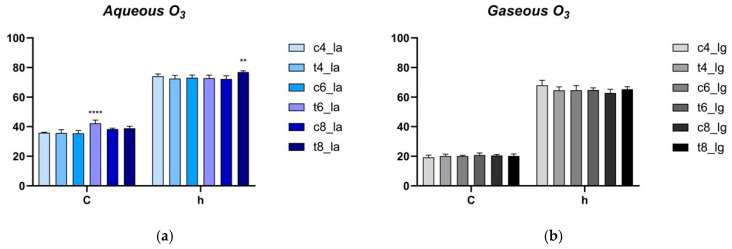
CIELab chroma and hue angle coordinates for the colorimetric measurements of samples after aqueous (**a**) and gaseous (**b**) ozone treatments. Samples nomenclature refers to that listed in Table 1. *p*-values for significant differences between control and treated samples are summarized with ** *p* < 0.01; **** *p* < 0.0001.

The color of lupin samples was investigated as one of the major quality attributes considered by the consumers, to evaluate a potential detrimental effect of the applied treatments. The measurement of CIELab coordinates (L*, lightness; a*, redness; b*, yellowness; C, Chroma; h, hue angle) was carried out on uninoculated lupin beans, subjected to either aqueous ozone treatments analogous to inoculated ones. In Figure 6 chroma and hue angle results of aqueous ozone (Figure 6a) and gaseous ozone (Figure 6b) treatments, respectively, are reported.

The results highlighted significant differences between control and treated samples only for a couple of samples exposed to water treatment. In fact, the samples treated with aqueous O_3_ for 6 h exhibited an appreciable increase in the chroma value, which passed from 35.44 ± 1.95 to 42.26 ± 2.13, while an increase in the hue angle from 62.87 ± 2.49 to 65.40 ± 1.64 was observed after 8 h of aqueous O_3_ treatment (Figure 6a).

Notably, no significant differences were perceived among the samples that underwent gaseous ozone treatment and in most aqueous O_3_ treatments. The ΔE analysis confirmed this result, showing that only the aqueous O_3_ treatment caused an appreciable change in the overall color appearance of the samples (Table 2). However, also in this case, only the 6 h treatment highlighted a distinct difference between samples (ΔE > 3), as could be argued from the raw data of CIELab coordinates (Table S1). This result is particularly important since it demonstrates a very low impact of the treatment on the overall appearance of lupin beans.

## 4. Discussion

The safety of lupin-based foods is primarily compromised by fungal contamination (PHO-A) and endogenous toxic compounds (QAs) [25]. Therefore, in this work, the efficacy of O_3_ was evaluated against PHO-A, QAs, and mold growth, to explore possible strategies for reducing the associated risk.

Concerning the effects of O_3_ on PHO-A, the results obtained are noteworthy since the starting concentration was significantly higher than the LOD (1.0 ppm). Moreover, given the high chemical stability of PHO-A, a 20% reduction is a significant result, which can help decrease contamination levels. These results are only partially in agreement with published data. As evidenced by some authors, the efficacy of ozone as an anti-mycotoxin treatment does not necessarily increase by extending the treatment duration [26]. Particularly, oxidation can be influenced by different factors, such as temperature, pH, and pressure, as well as the food matrix and the mycotoxin distribution [13]. Although other studies have agreed on the idea that extended ozonation treatments can lead to increased reductions in mycotoxins content [27], in our study the treatment efficacy was stable during time, probably because of the deep inoculation of *D. toxica* and the consequent presence of PHO-A underneath the seeds surface. In fact, as shown for the aqueous ozonation [28], the effectiveness of ozone is limited to the surface of the product. Based on our data, gaseous O_3_ treatments did not have an efficacy against PHO-A. These results contradict numerous research in the literature that claim not only a greater efficacy of gaseous O_3_ but also a stronger detoxifying activity compared to aqueous O_3_ treatments [29].

Some studies have confirmed the superior efficacy of aqueous O_3_ against mycotoxins, especially where some gaseous O_3_ yielded no appreciable results [30,31]. In this case, the aqueous medium may have acted as a more efficient carrier, facilitating deeper ozone penetration into lupins outer layers. Compared with the existing evidence concerning the detoxification of mycotoxins by ozone, these results suggest that this technology acts by directly degrading the mycotoxin through its oxidizing effects. The main mechanism proposed for mycotoxins inactivation by means of ozone is the reactivity against exposed unsaturated chemical bonds, and therefore the most susceptible molecules are Aflatoxins B1 and G1, and trichothecenes [32]. Consequently, the observed result could be attributable to the higher resistance of PHO-A compared to other mycotoxins, and its different chemical structure, including its hexapeptide formation and modifications, which was frequently demonstrated as greatly stable even when subjected to strong detoxification treatments [32,33]. The observed significant reduction in PHO-A levels following ozone treatment represents a crucial result, especially considering the current lack of reported physical or chemical detoxification methods for this mycotoxin in the literature, that can help decrease the decontamination level below the regulatory threshold of 5 μg/kg. While O_3_ efficacy is very promising, the complex nature of its reactions with organic molecules makes resulting chemical structures and biological activities difficult to identify. Nevertheless, the ability of O_3_ demonstrated in PHO-A reduction makes it a valuable technology in ensuring lupin safety, evidencing the need for future studies elucidating PHO-A degradation pathways and assessing the reaction products safety for human health.

Considering the quantification of alkaloid species in lupin beans after O_3_ treatment, the results strongly agree with many studies where a decrease in alkaloids was observed in various plant materials, even though no evidence has been reported on QA. For instance, ozone treatments resulted in a significant reduction in pyrrolizidine alkaloids in oregano and indole alkaloids in chickpea grains [15,16]. No results have been reported in the literature on lupin beans directly exposed to ozone treatments, even though it has been proved that elevated concentrations of ozone stress the plant, increasing the concentrations of secondary metabolites, including alkaloids, by regulating the polyamines in the alkaloid pathway [34]. Moreover, because of the effect of ozone, it could not be excluded that other QA species, undergoing strong oxidation, could generate one of the species under investigation.

Similarly to what was described for PHO-A, while the reduction in QA levels can be considered a positive result, chemical structures and biological activities of their degradation products remain largely uncharacterized. In fact, O_3_ reaction with complex organic molecules often leads to the formation of numerous intermediate products, which remain difficult to identify and may possess different toxicological properties than the original compounds. However, despite this potential issue, the efficacy demonstrated in alkaloid reduction makes O_3_ a technology worth to be furtherly explored. Differently from the good potential shown by ozone in reducing PHO-A and QAs in lupin samples, microbiological investigations indicated that O_3_ treatments resulted in relatively minimal reduction in fungal growth. In this respect, the scientific literature on the efficacy of O_3_ on molds indicates that the effects can be inconsistent and frequently constrained due to several factors. Notably, while ozone can delay hyphae development and reduce sporulation, it does not consistently eradicate the infection, a condition that may elucidate the findings of the current study. Moreover, according to other authors, the effectiveness of O_3_ exhibited a high variability depending on the fungal species: for instance, certain *Aspergillus* species revealed different responses to treatments, with some displaying greater susceptibility than others [35,36]. Furthermore, many environmental conditions have been demonstrated to be potentially involved in O_3_ effectiveness in fungal inactivation [37].

PHO-A reduction was inconsistent with fungal inhibition. Thus, the observed 19.83, 19.44 and 20.40% reductions in PHO-A are highly due to a direct chemical degradation of the already formed toxin. In fact, PHO-A presents different disulphide bonds and unsaturated carbon-carbon bonds, as well as aromatic or heterocyclic structures, which are potential sites for oxidative attack by ozone [38]. Consequently, further steps could include not only the evaluation of the fungal load after the incubation time, but also the influence of the treatment on mycelia growth and spore germination, and a screening of detoxification reaction intermediates. Finally, the color of the lupin beans was evaluated as a quality attribute after O_3_ treatments to determine if these treatments might affect their appearance and, consequently, their acceptability to the consumer. Particularly, ΔE measurement demonstrated no perceivable differences in any of the samples undergone to gaseous treatments and in most of aqueous treatments. This stability is particularly noteworthy, given the strong oxidative potential of O_3_. Therefore, the outcomes of this study demonstrate that the treatments targeted the contaminants (PHO-A and QAs) without inducing significant browning of the seeds. This behavior is quite common as previous studies evidenced the minimal impact of ozone treatments on the color attributes of legumes; particularly, no significant variations were observed on dried green beans or chickpeas [15,39]. Consequently, it can be argued that O_3_ can be suitable for the potential industrial scale-up for treatments of legume seeds and seems to fit the detoxification of lupin beans.

## 5. Conclusions

This study aimed at evaluating the effectiveness of different ozone treatments on the main safety-related concern of lupin seeds, to upgrade their value both as ready-to-eat products, and as new plant-based protein sources.

The obtained results suggest an interesting activity of aqueous ozone that was able to produce a 19.83, 19.44, and 20.40% reduction in PHO-A concentration (Figure 3). This result is noteworthy considering that this mycotoxin is particularly resistant to detoxification procedures. Simultaneously, a reduction in QA from 22.17% and up to 71.86% was observed after O_3_ aqueous treatments. However, specific toxicological data on the ozone-induced degradation products of PHO-A and QAs in lupin beans are currently scarce. Therefore, a comprehensive assessment of the safety of ozone-treated lupin products for human consumption would ideally require the identification and subsequent toxicological evaluation of these specific by-products. Interestingly, the treatments minimally impacted the color properties of lupin seeds, demonstrating how ozone could be a suitable technology in lupin production process.

In addition, gaseous ozone treatments showed lower effectiveness although, in some cases, they demonstrated a potential that could be explained by further studies. In fact, most of the available literature agrees in attributing gaseous ozone a higher efficacy than aqueous ozonation.

Based on the results achieved, this study proposes an innovative solution to mitigate the risk associated with PHO-A and QA in lupin beans. Ozone can be applied directly in the industrial washing tanks, without major changes in the equipment. This solution also improves the effectiveness of the washing procedures. Considering the high stability of PHO-A, our results can be considered useful for mitigating the effect of PHO-A and QA compounds on health, although additional evaluation of the toxicity of these compounds after ozone treatments are recommended. Future research should also address the possible impact of ozone treatments on lupin nutrients and on the bioactive potential of this legume.

## Figures and Tables

**Table 1:** Description of the samples prepared for aqueous and gaseous ozone treatments.

Treatment	Duration (h)	Sample	Description
Aqueous O_3_	4	t4_la	Lupin beans subjected to 4 h aqueous treatment with an O_3_ concentration of 7.00 ± 0.01 ppm.
		c4_la	Control sample t4_la.
	6	t6_la	Lupin beans subjected to 6 h aqueous treatment with an O_3_ concentration of 7.00 ± 0.01 ppm.
		c6_la	Control sample t6_la.
	8	t8_la	Lupin beans subjected to 8 h aqueous treatment with an O_3_ concentration of 7.00 ± 0.01 ppm
		c8_la	Control sample t8_la.
Gaseous O_3_	4	t4_lg	Lupin beans subjected to 4 h gaseous treatment with an O_3_ concentration of 7.00 ± 0.01 ppm.
		c4_lg	Control sample t4_lg.
	6	t6_lg	Lupin beans subjected to 6 h gaseous treatment with an O_3_ concentration of 7.00 ± 0.01 ppm.
		c6_lg	Control sample t4_lg.
	8	t8_lg	Lupin beans subjected to 8 h gaseous treatment with an O_3_ concentration of 7.00 ± 0.01 ppm
		c8_lg	Control sample t8_lg.

**Table 2:** Global color difference (ΔE) between control and treated samples.

Treatment	Duration (h)	ΔE
Aqueous O_3_	4	2.32
	6	7.77
	8	3.19
Gaseous O_3_	4	1.14
	6	1.16
	8	1.21

## Data Availability

The original contributions presented in the study are included in the article/Supplementary Material, further inquiries can be directed to the corresponding author.

## Supplementary Materials

The following supporting information can be downloaded at: https://www.mdpi.com/article/10.3390/foods15020326/s1, Table S1. *L**, *a**, *b**, C, h coordinates evolution of lupin samples following different ozonation treatments. Samples nomenclature refers to that listed in Table 1. *p*-values for significant differences between control and treated samples are summarized with * *p* < 0.05; ** *p* < 0.01; *** *p* < 0.001; **** *p* < 0.0001.
