# Supplementary material for: Phomopsin-A and Quinolizidine Alkaloids Concentrations in Lupinus albus Seeds: Effect of Aqueous and Gaseous Ozone Application
Source: Foods. 2026 Jan 15;15(2):326. doi: 10.3390/foods15020326 (PMC12840407; doi:10.3390/foods15020326)
Supplement: Supplementary file 1 [file foods-15-00326-s001.zip › Table S1 - Foods.pdf]

Table S1. *L**, *a**, *b**, C, h coordinates evolution of lupin samples following different ozonation treatments. Samples nomenclature refers to that listed in Table 1. p-values for significant differences between control and treated samples are summarized with * $p<0.05$; ** $p<0.01$; *** $p<0.001$; **** $p<0.0001$.

Treatment	*L**	*a**	*b**	C	h
c4_la	60.14 ± 0.48	9.76 ± 0.88	34.47 ± 0.52	35.83 ± 0.34	74.19 ± 1.55
t4_la	58.03 ± 1.52	10.66 ± 0.98	34.09 ± 2.41	35.74 ± 2.20	72.55 ± 2.18
c6_la	61.06 ± 1.76	10.28 ± 1.19	33.90 ± 1.94	35.44 ± 1.95	73.12 ± 1.87
t6_la	57.33 ± 0.55	12.42 ± 0.81	40.37 ± 2.47	42.26 ± 2.13****	72.82 ± 2.03
c8_la	59.36 ± 1.45	11.66 ± 1.43	36.48 ± 0.67	38.32 ± 0.67	72.28 ± 2.13
t8_la	59.42 ± 0.52	8.77 ± 0.54	37.83 ± 1.39	38.84 ± 1.36	76.94 ± 0.88**
c4_lg	60.51 ± 1.36	7.79 ± 0.52	17.34 ± 1.58	19.27 ± 1.44	68.07 ± 3.27
t4_lg	60.70 ± 1.37	8.32 ± 0.58	18.33 ± 1.23	20.13 ± 1.22	68.07 ± 2.36
c6_lg	59.74 ± 1.52	8.60 ± 1.01	17.85 ± 0.67	20.09 ± 0.57	64.72 ± 3.18
t6_lg	60.89 ± 1.26	8.58 ± 0.82	17.75 ± 1.31	20.77 ± 1.47	64.84 ± 1.46
c8_lg	60.41 ± 1.57	9.22 ± 0.51	18.84 ± 1.07	20.51 ± 0.72	62.87 ± 2.49
t8_lg	60.91 ± 1.25	8.17 ± 0.27	18.47 ± 1.32	20.23 ± 1.32	65.40 ± 1.64
